# Molecular Characterization of 87 Functional Genes in Wheat Diversity Panel and Their Association With Phenotypes Under Well-Watered and Water-Limited Conditions

**DOI:** 10.3389/fpls.2019.00717

**Published:** 2019-06-04

**Authors:** Maria Khalid, Fakiha Afzal, Alvina Gul, Rabia Amir, Abid Subhani, Zubair Ahmed, Zahid Mahmood, Xianchun Xia, Awais Rasheed, Zhonghu He

**Affiliations:** ^1^Atta-ur-Rehman School of Applied Biosciences (ASAB), National University of Science and Technology (NUST), Islamabad, Pakistan; ^2^Institute of Crop Sciences, National Wheat Improvement Centre, Chinese Academy of Agricultural Sciences (CAAS), Beijing, China; ^3^Barani Agriculture Research Institute (BARI), Chakwal, Pakistan; ^4^Crop Science Institute, National Agricultural Research Centre, Islamabad, Pakistan; ^5^International Maize and Wheat Improvement Centre (CIMMYT), CAAS, Beijing, China; ^6^Department of Plant Sciences, Quaid-i-Azam University, Islamabad, Pakistan

**Keywords:** drought tolerance, functional markers, kompetitive allele-specific PCR markers, synthetic-derivatives, marker-trait association

## Abstract

Modern breeding imposed selection for improved productivity that largely influenced the frequency of superior alleles underpinning traits of breeding interest. Therefore, molecular diagnosis for the allelic variations of such genes is important to manipulate beneficial alleles in wheat molecular breeding. We analyzed a diversity panel largely consisted of advanced lines derived from synthetic hexaploid wheats for allelic variation at 87 functional genes or loci of breeding importance using 124 high-throughput KASP markers. We also developed two KASP markers for water-soluble carbohydrate genes (*TaSST-D1* and *TaSST-A1*) associated with plant height and thousand grain weight (TGW) in the diversity panel. KASP genotyping results indicated that beneficial alleles for genes underpinning flowering time (*Ppd-D1* and *Vrn-D3*), thousand grain weight (*TaCKX-D1, TaTGW6-A1, TaSus1-7B*, and *TaCwi-D1*), water-soluble carbohydrates (*TaSST-A1*), yellow-pigment content (*Psy-B1* and *Zds-D1*), and root lesion nematodes (*Rlnn1*) were fixed in diversity panel with frequency ranged from 96.4 to 100%. The association analysis of functional genes with agronomic and biochemical traits under well-watered (WW) and water-limited (WL) conditions revealed that 21 marker-trait associations (MTAs) were consistently detected in both moisture conditions. The major developmental genes such as *Vrn-A1, Rht-D1*, and *Ppd-B1* had the confounding effect on several agronomic traits including plant height, grain size and weight, and grain yield in both WW and WL conditions. The accumulation of favorable alleles for grain size and weight genes additively enhanced grain weight in the diversity panel. Graphical genotyping approach was used to identify accessions with maximum number of favorable alleles, thus likely to have high breeding value. These results improved our knowledge on the selection of favorable and unfavorable alleles through unconscious selection breeding and identified the opportunities to deploy alleles with effects in wheat breeding.

## Introduction

Insight into genetic loci that have been selected during modern wheat breeding is of significant importance to understand the phenotypic variation in modern wheat cultivars. This will enable wheat breeding to transit to knowledge-based activity and will ultimately improve the rate of genetic progress in wheat (Li H. et al., 2018). The major limitations, however, are the: (i) unavailability of full spectrum of functional genes involved in wheat adaptability, (ii) sequencing resources in large diversity panels of wheat, and (iii) high-throughput genotyping platforms for gene diagnostics. Gene discovery and map-based gene cloning in wheat has lagged behind other crops, such as rice and maize, due to the slow progress in developing sequence resources due to the large and complex wheat genome. Consequently, gene cloning in the Triticeae (wheat and its related species) was reliant to some extent on comparative genomics approaches with other grass genomes due to the high collinearity and genetic organization among grass genomes (Li H. et al., 2018). Until now, more than 150 functional markers are available for important genes in wheat (Liu et al., 2012), and were subsequently converted into high-throughput KASP assays (Rasheed et al., 2016a). Such as, genes related to grain size and weight (Nadolska-Orczyk et al., 2017), developmental traits like photoperiod response and vernalization (Kamran et al., 2014), and end-use quality. More specifically genes for grain size and weight including *TaTGW6* (Hanif et al., 2015; Hu et al., 2016), *TaCwi-*A1 (Ma et al., 2012), *TaSus2-2B* (Jiang et al., 2011), *TaSus2-2A, TaSus1-7A* (Hou et al., 2014), *TaGW2-6A, 6B* (Su et al., 2011; Yang et al., 2012; Qin et al., 2014), *TaCKX6-*D1 (Zhang et al., 2012), *TaSAP1-A1* (Chang et al., 2013), *TaGS-D1* (Zhang et al., 2014; Ma et al., 2016), and *TaGASR-*A1 (Dong et al., 2014) have been cloned in wheat using comparative genomics approaches.

The favorable alleles of some genes are not very well surveyed in wheat germplasm from different regions and genetic backgrounds. Such studies hold promise to find opportunities to deploy specific favorable alleles using molecular markers. Similarly, those genes favoring adaptability of wheat under drought stress are extremely important for breeding purpose (Zhang et al., 2008). Drought is currently leading threat for global food security which is getting more severe with climate change following drier environment (Acuna-Galindo et al., 2014). Drought affects wheat at all developmental stages, including reproductive and grain filling stages which are the most sensitive phases of crop development, resulting in significant yield loss (Li C. et al., 2018). Therefore, deployment of genes favoring drought adaptability in selection breeding is a key strategy for sustainable wheat production (Thapa et al., 2018).

Synthetic hexaploid wheat (SHW) is an excellent source to transfer genetic variation for cultivar improvement. It combines genes from wild ancestor *Aegilops tauschii* (Coss.) and tetraploid wheat (*Triticum turgidum* L.), which improves bread wheat through the identification and introgression of useful genes via synthetic derivatives (SYN-DER) or advanced derivatives synthetic backcross derived lines (SBLs). It has been concluded during past few years that 30% yield advantage under drought stress could be attributed to the use of SHW (Tang et al., 2015). The favorable alleles associated with yield related traits retained in SBLs are the reason of yield benefits in SHW (McIntyre et al., 2014). However, the proportion of favorable alleles of major genes retained in SYN-DER is largely unknown, and analysis of allelic effects of these genes could be helpful in deployment of certain genes related to developmental traits and drought adaptability (Afzal et al., 2017).

The objectives of current study were to (i) analyze the allelic variations of functional genes underpin developmental traits, end-use quality, disease resistance, drought tolerance and agronomic traits, (ii) assess the allelic effects of these genes in SYN-DER diversity panel under well-watered (WW) and water-limited conditions (WL), and (iii) identify the genotypes with maximum favorable alleles or haplotypes under drought stress conditions.

## Materials and Methods

### Germplasm and Phenotypic Evaluation

A diversity panel of 213 advanced lines derived from synthetic hexaploid wheats (SHWs) and elite bread wheat cultivars was used in the present study (Supplementary Table S1), comprising 42 bread wheat genotypes and 171 SYN-DERs. SYN-DER lines were developed by crossing synthetic lines with advanced lines and improved cultivars from CIMMYT and Pakistan.

The diversity panel was planted in the field in Barani Agriculture Research Institute, Chakwal (72.8°E, 32.8°N; 575 m a.m.s.l.) and National Agriculture Research Centre, Islamabad, Pakistan (73.04°E, 33.68°N; 620 m a.m.s.l.) following an alpha lattice design. Each genotype of two 2-m long rows was sown with two replications. Thirty viable seeds were sown for each row with the help of small plot grain drill for each row. Physiological, agronomic and biochemical traits have been measured according to our previous publication (Afzal et al., 2017).

For both well-water treatment and water limited treatment, inter-row spacing of 30 cm has been maintained. This cropping season was screened for 2 years in two environmental conditions. One is well water condition (WW) and the other is water limited condition (WL) which is semiarid and rainfed area lies at start of Potohwar plateau. For well-watered condition, three irrigations were given and soil moistures have been maintained until harvest. For water limiting conditions, polyethylene tunnel is used to provide shelter from precipitation in water limiting environment. The water seepage was avoided by 1 m deep ditch surrounding the tunnel and irrigation was stopped at the end of tillering stage.

Phenotypic traits recorded at each location included chlorophyll content index (CCI), total chlorophyll (Chl), canopy temperature (CT), number of grains per spike (GS), grain yield (GY), plant height (PH), proline, relative water contents (RWC), shoot dry weight (SDW), shoot fresh weight (SFW), spike length (SL), superoxide dismutase (SOD), sugar contents (Sugar), thousand grain weight (TGW), and tillers per plant (TP). Specific suffixes were provided to each trait according to the water treatment, e.g., GY_WW_ (GY in well-watered treatment) and GY_WL_ (GY in water-limited treatment). The detailed phenotyping methodology for biochemical traits is provided earlier (Afzal et al., 2019).

### Genotyping

DNA was extracted from all genotypes using a modified CTAB method (Dreisigacker et al., 2013). Allele-specific KASP markers for 87 different loci were used (Supplementary Table S2). The primer sequences and amplification conditions of each gene are described in Supplementary Table S2. KASP assay to genotype targeting SNP in SYN-DER was developed following standard KASP guidelines. Briefly, primers were designed carrying standard FAM tail (5′-GAAGGTGACCAAGTTCATGCT-3′) and HEX tail (5′-GAAGGTCGGAGTCAACGGATT-3′), with targeting SNP at the 3′end. Primer mixture included 46 μl ddH_2_O, 30 μl common primer (100 μM) and 12 μl of each tailed primer (100 μM). Assays were tested in 384-well format and set up as 5 μl reaction [2.2 μl DNA (10–20 ng/μl), 2.5 μl of 2XKASP master mixture and 0.056 μl primer mixture]. PCR cycling was performed using following protocol: hot start at 95°C for 15 min, followed by ten touchdown cycles (95°C for 20 s; touchdown 65°C–1°C per cycle 25 s) further followed by 30 cycles of amplification (95°C for 10 s; 57°C for 60 s). Extension step is unnecessary as amplicon is less than 120 bp. Plate was read in BioTek H1 system and data analysis was performed manually using Klustercaller software (version 2.22.0.5; LGC Hoddesdon, United Kingdom).

### Development of KASP Assays for *TaSST* Genes

Two KASP assays were developed for *TaSST-D1* and *TaSST-A1* genes underpin water-soluble carbohydrates (Dong et al., 2016). One KASP marker was developed for SNP A/G at position 1093 bp corresponding to CAPS marker WSC7D of *TaSST-D1*. Second KASP marker was developed for two neighboring SNPs GTT/ATA at positions 438 and 440 bp in *TaSST-A2* gene. The results were compared to wheat cultivars in our previous publication (Dong et al., 2016).

### Statistical Analysis

Association analysis was performed in TASSEL version 5.0 using mixed linear model (MLM) which takes into account the K-PC model (Bradbury et al., 2007). This diversity has been genotyped with 90K SNP array (Afzal et al., 2019) and 500 unlinked SNP markers representing all 21 wheat chromosomes having minor allele frequency (MAF) value ranging from 0.3 to 0.5 were used for principal component analysis (PCA) and kinship information in TASSEL version 5.0. Both kinship matrix and first five principal components from PCA were used as co-variate to improve the statistical power. For the markers significantly associated with phenotypes in TASSEL, the student’s *t*-test and Kruskal–Wallis test were further used to compare allelic effect means with threshold probability *P* < 0.01 for two and more haplotypes in a given trait, respectively.

## Results

In total, 124 KASP markers were used to identify alleles at 87 loci in SYN-DER diversity panel. The results described here are presented for the alleles or haplotypes as shown in Table 1 at each locus, instead of presenting results for individual markers. Because in some cases, several KASP assays were used to identify multiple alleles at single locus. The alleles at 30 loci were fixed with relatively high frequency ranging from 95 to 100% in the diversity panel (Table 2). Such markers were not used for marker-trait association analysis. Those markers which were used for MTAs are shown in Table 3. In total, 58 loci showed allelic variation with minor allele frequency >5%. These loci underpin plant stature (*n* = 2), flowering time (*n* = 9), grain size and weight (*n* = 11), drought adaptability (*n* = 5), end-use quality (*n* = 18), grain color and dormancy (*n* = 7), and disease resistance (*n* = 6) (Table 1).

**TABLE 1:** Allele frequency of functional genes in the diversity panel derived from synthetic hexaploid wheat.

**Group**	**Gene**	**Mode^a^**	**Mode frequency**	**Allele/haplotype**	**Number**	**Frequency (%)**
Adaptability	*Rht-B1*	*Rht-B1a*	106	*Rht-B1a*	106	50.96
				*Rht-B1b*	97	46.63
	*Rht-D1*	*Rht-D1a*	161	*Rht-D1a*	161	77.40
				*Rht-D1b*	45	21.63
	*1BL.1RS*	*1B1R-*	136	1B1R-	136	65.38
				1B1R+	55	26.44
Flowering time	*Ppd-A1*	*Ppd-A1a*	127	*Ppd-A1a*	127	61.06
				*Ppd-A1a (1027)*	1	0.48
				*Ppd-A1a (1117)*	16	7.69
				*Ppd-A1b*	64	30.77
	*Ppd-B1*	*Ppd-B1a*	157	*Ppd-B1a*	157	75.48
				*Ppd-B1a* (Chayenne-type)	10	4.81
				*Ppd-B1a* (Sonoro)	41	19.71
	*Vrn-A1* (Exon7)	Claire-type	155	Claire-type	155	74.52
				Hereward-type	39	18.75
	*Vrn-A1*	*Vrn-A1w* (Jagger)	91	*Vrn-A1a*	6	2.88
				*Vrn-A1s*	47	22.60
				*Vrn-A1w*	18	8.65
				*Vrn-A1w* (2147)	46	22.12
				*Vrn-A1w* (Jagger)	91	43.75
	*Vrn-B1*	*Vrn-B1a*	179	*Vrn-B1a*	179	86.06
				*Vrn-B1b*	11	5.29
				*Vrn-B1c*	2	0.96
				*vrn-B1*	4	1.92
	*Vrn-D1*	*Vrn-D1 (S)*	185	*Vrn-D1*	185	88.94
				*vrn-D1*	23	11.06
	*TaBradi2g14790*	Deletion	96	Insertion	86	41.35
				Deletion	96	46.15
	*TaMOT1-D1*	Wild-type	164	Ria-type	8	20.85
				Wild-type	164	78.85
	*PRR73-A1*	*Hap-I*	120	Hap-I	120	57.69
				Hap-II	77	37.02
	*PRR73-B1*	*Hap-I*	196	Hap-I	196	94.23
				Hap-II	10	4.81
Yield components	*TaGS-D1*	*TaGS-D1a*	171	*TaGD-D1b*	29	13.94
				*TaGS-D1a*	171	82.21
	*TaCwi-A1*	*TaCwi-A1a*	175	*TaCwi-A1a*	175	84.13
				*TaCwi-A1b*	30	14.42
	*TaGASR-A1*	*H1g*	185	*H1c*	17	8.17
				*H1g*	185	88.94
	*TaSus2-2A*	*Hap-A*	115	*Hap-A*	115	55.29
				*Hap-G*	85	40.87
	*TaSus1-7A*	*TaSus-7A-1*	180	*TaSus-7A-3*	17	8.17
				*TaSus-7A-1*	180	86.54
				*TaSus-7A-2*	8	3.84
	*TaGS-A1*	*TaGS5-A1a*	166	*TaGS5-A1a*	166	79.81
				*TaGS5-A1b*	40	19.23
	*TaGW2-6B*	*Hap-I*	128	*Hap-I*	128	61.54
				*Hap-II*	80	38.46
	*TaGS2-A1*	*TaGS2-A1b*	133	*TaGS2-A1a*	61	29.33
				*TaGS2-A1b*	133	63.94
	*TEF-7A*	*Hap-7A-1*	137	*Hap-7A-3*	50	24.02
				*Hap-7A-2*	20	9.62
				*Hap-7A-1*	137	65.87
	*TaGS1a*	*Hap-II*	121	Hap-I	75	36.06
				Hap-II	121	58.17
Drought adaptability	*1fehw3*	*Kauz-type*	104	Kauz-type	104	50.00
				Westonia-type	99	47.60
	*TaCwi-A1*	*Hap-4A-C*	123	Hap-4A-C	123	59.13
				Hap-4A-T	68	32.69
	*COMT-3B*	*COMT-3Ba*	115	3Bb	86	41.35
				3Ba	115	55.29
	*TaSST-4D*	*4Db*	113	4Da	80	38.46
				4Db	113	54.33
	*Dreb1*	*TaDREB-B1b*	104	*TaDREB-B1a*	86	41.35
				*TaDREB-B1b*	104	50.00
Grain color and dormancy	*TaSdr-B1*	*TaSdr-B1b*	177	*TaSdr-B1a*	23	11.06
				*TaSdr-B1b*	177	85.10
	*Vp1-B1*	*Vp1-B1c*	144	*Vp1-B1a*	62	29.81
				*Vp1-B1b*	2	0.96
				*Vp1-B1c*	144	69.23
	*PHS1*	RioBlanceo type	158	NW97S186 type	38	18.27
				RioBlanceo type	158	75.96
	*TaMFT-A1*	Zen-type	172	Zen-type	177	85.00
				Jagger-type	31	15.00
	*Tamby10-A1*	*R-A1a*	108	*R-A1a*	108	51.92
				*R-A1a (Norin)*	2	0.96
				*R-A1b*	76	36.54
	*Tamyb10-B1*	*R-B1b*	195	*R-B1a*	12	5.77
				*R-B1b*	195	93.75
	*Tamyb10-D1*	*R-D1a*	178	*R-D1a*	178	85.58
				*R-D1b*	28	13.46
End-use quality	*Glu-A1*	Ax2^*^	84	*Ax1*	57	27.40
				Ax2^*^	84	40.38
				*AxNull*	67	32.21
	*Glu-D1*	5+10	105	2+12	94	45.19
				5+10	105	50.48
	*Pina-D1*	*Pina-D1b*	170	*Pina-D1a*	32	15.38
				*Pina-D1b*	170	84.62
	*Pinb-D1*	*Pinb-D1b*	132	*Pinb-D1a*	58	27.88
				*Pinb-D1b*	132	63.46
	*Pinb2*	*Pinb-B2a*	119	*Pinb-B2a*	119	57.21
				*Pinb-B2b*	83	39.90
	*PPO-A1*	*Ppo-A1a*	83	*Ppo-A1a*	75	36.06
				*Ppo-A1b*	40	19.23
	*PPO-D1*	*Ppo-D1b*	105	*Ppo-D1a*	93	44.71
				*Ppo-D1b*	105	50.48
	*Ppo-B2*	*Ppo-B2c*	177	*Ppo-B2a,b*	24	11.54
				*Ppo-B2c*	177	85.10
	*PPO-D2*	*Ppo-D2b*	188	*Ppo-D2a*	19	9.13
				*Ppo-D2b*	188	90.38
	*Psy-A1*	*Psy-A1a*	123	*Psy-A1a*	123	59.13
				*Psy-A1b*	78	37.50
	*Psy-D1*	*Psy-D1a*	197	*Psy-D1a*	197	94.71
				*Psy-D1g*	2	0.96
	*Wx-B1*	*Wx-B1a*	145	*Wx-B1a*	145	69.71
				*Wx-B1b*	63	30.29
	*Pds-B1*	*TaPds-B1a*	190	*TaPds-B1a*	190	91.35
				*TaPds-B1b*	11	5.29
	*Pod-A1*	*TaPod-A1a*	203	*TaPod-A1a*	203	97.60
				*TaPod-A1b*	1	0.48
	*LYC-B1*	*TaLyc-B1b*	132	*TaLyc-B1a*	59	28.37
				*TaLyc-B1b*	132	63.46
	*TaZds-A1*	*TaZds-A1a*	112	*TaZds-A1a*	112	53.85
				*TaZds-A1b*	73	35.10
	*WBM*	*wbm-*	170	*wbm+*	34	16.35
				*wbm-*	170	81.73
	*NAM-A1*	*NAM-A1c*	106	*NAM-A1a*	102	49.04
				*NAM-A1c*	106	50.96
Disease resistance	*Lr34*	*Lr34+*	113	*Lr34+*	113	54.33
				*Lr34-*	90	43.27
	*Lr14a*	*Lr14a-*	135	*Lr14a+*	71	34.13
				*Lr14a-*	135	64.90
	*Sr2*	*Sr2-*	201	*Sr2+*	7	3.37
				*Sr2-*	201	96.63
	*Lr21*	*Lr21-*	198	*Lr21+*	10	4.81
				*Lr21-*	198	95.19
	*SbmP*	*Sbmp-*	188	*Sbmp+*	20	9.62
				*Sbmp-*	188	90.38
	*Cre8*	*Cre8-*	146	*Cre8+*	47	22.60
				*Cre8-*	146	70.19

*^a^Mode, Major allele/allele with the highest frequency.*

**TABLE 2:** Alleles of functional genes completely or pre-dominantly fixed in the diversity panel derived from synthetic hexaploid wheat.

**Group**	**Gene**	**Allele**	**Number**	**Frequency**	**Effect of major allele**
Flowering time	*Ppd-D1*	*Ppd-D1a*	203	99.60	Early flowering
	*Vrn-B3*	*vrn-B3 (CS)*	203	97.60	Delay flowering
	*Vrn-D3*	*Jagger-type*	206	99.04	Short vernalization requirement
	*TaELF3-B1*	*Cadenza-type*	194	93.27	Delay flowering
	*TaELF3-D1*	*Wild-type*	203	96.0	Delay flowering
Yield components	*TaSus2-2B*	*Hap-L*	208	100.00	Low TGW
	*TaGW2-6A*	*Hap-6A-G*	202	97.12	Low TGW
	*TaCKX-D1*	*TaCKX-D1a*	186	98.42	High TGW
	*TaTGW6-A1*	*TaTGW6-TaTGW6A1a*	204	98.08	High TGW
	*TaMoc-A1*	*Hap-L*	204	98.08	Low grain number
	*TaSus1-7B*	*Hap-T*	195	97.75	High TGW
	*TaTGW6-4A*	*TaTGW6-b*	201	96.63	Low TGW
	*TaGS2-B1*	*Hap-A*	187	98.90	NA
	*TaCwi-D1*	*Hap-5D-C*	206	99.04	High TGW
Drought adaptability	*AWN_BWc8266_5AL*	*Presence*	204	98.08	AWN present
	*TaSST-A1*	*A2a*	196	94.23	High WSC
End-use quality	*PPOA2b_230*	*Ppo-A2a,c*	206	99.04	NA
	*Psy-B1*	*Psy-B1a or b*	204	98.08	Low YPC
	*Zds-D1*	*TaZds-D1b*	205	98.56	Low YPC
	*Lox-B1*	*Lox-B1a*	188	90.38	High LOX
	*PPO-A2*	*PPO-A2a*	206	99.52	NA
Disease resistance	*Fhb1*	*Fhb1-*	205	98.56	FHB Susceptible allele
	*Sr36/Pm6*	*Sr36-*	208	100.00	Stem rust susceptible allele
	*Lr37*	*Lr37-*	208	100.00	Leaf rust susceptible allele
	*Lr9*	*Lr9-*	208	100.00	Leaf rust susceptible allele
	*Lr67*	*Lr67-*	208	100.00	Leaf rust susceptible allele
	*Lr68*	*Lr68-*	208	100.00	Leaf rust resistance allele
	*Lr47*	*Lr47-*	191	91.83	Leaf rust susceptible allele
	*Rlnn1*	*Rlnn1+*	202	97.12	Root lesion nematode resistance allele
	*CU8*	*CU8-*	208	100.00	Eye spot susceptible allele

**TABLE 3:** Marker-trait associations in the diversity panel derived from synthetic hexaploid wheat under well-watered and water-limited conditions.

			**Well-watered**			**Water-limited**		
**Trait**	**Marker**	**MAF**	***P***	***R^2^***	**Estimate**	***P***	***R^2^***	**Estimate**
DH	*TaCwi-4A*		3.2E-03	0.022	–9.20	1.32E-04	0.069	11.10
DH	*Vrn-A1*		4.6E-03	0.019	0.99	4.06E-04	0.059	–11.91
GpS	*Rht-B1*		3.6E-03	0.021	3.60	0.0230	0.025	–1.47
GY	*Rht-D1*		3.9E-03	0.020	0.48	0.0204	0.026	16.11
GY	*TaGASR-A1*		3.6-E03	0.020	–6.13	0.0128	0.029	–1.51
GY	*Vrn-A1*		1.6E-03	0.027	16.48	0.0064	0.035	1.30
PH	*Rht-D1*		1.9E-03	0.026	–2.37	7.23E-04	0.054	4.59
PH	*TaCwi-A1*		1.0E-04	0.048	–3.65	0.0364	0.020	–3.70
PH	*TaSST-4D*		1.6E-03	0.027	2.99	0.0058	0.036	4.07
SL	*TaSus1-7A*		3.3E-04	0.021	–5.53	0.0251	0.024	–1.09
SOD	*TaSus1-7B*		1.5E-03	0.027	–1.02	0.0051	0.037	–1.66
Soluble Sugar	*COMT-3B*		9.0E-03	0.032	–2.96	0.0339	0.021	–12.95
Soluble Sugar	*TaCwi-A1*		3.9E-02	0.020	33.0	0.0034	0.040	24.98
TGW	*TaCwi-4A*		1.6E-02	0.028	–2.39	0.028	0.023	–2.16
TGW	*Rht-D1*		5.0E-03	0.036	3.20	8.32E-04	0.053	3.82
TGW	*TaSST-4D*		1.6E-02	0.027	–2.22	0.020	0.025	–2.71
TGW	*TaCwi-A1*		9.35E-03	0.021	3.047	5.1E-03	0.037	3.69
TGW	*TaGS1a*		2.1E-04	0.043	–2.67	5.07E-04	0.057	–3.23
TGW	*Ppd-B1*		5.1E-03	0.036	–3.20	0.027	0.023	–2.43
TGW	*Vrn-A1*		2.32E-04	0.063	4.13	4.41E-04	0.058	3.98
TN	*PRR73-A1*		2.1E-02	0.025	0.388	0.011	0.030	–0.77

*MAF, Minor allele frequency; *P*, *P*-values; *R^2^*, Phenotypic variation explained by marker; Estimate, Allelic effect of minor allele.*

### Allele Fixation at Loci of Breeding Interest in SYN-DER

The alleles present in more than 95% of the accessions were referred to the fixed or highly selected alleles and are mentioned in Table 2. Alleles were referred to “favorable alleles” if positively associated with the improved phenotype in literature and referred to “unfavorable alleles” if the allelic effect is not favorable for improved phenotype. At *Ppd-D1* locus, the photo-period insensitive allele, *Ppd-D1a*, was fixed with frequency of 99.6%. Similarly, Jagger-type allele at *Vrn-D3* gene was present in 99.04% accessions which is associated with short-vernalization requirement. The Cadenza-type and wild-type alleles associated with delayed flowering were identified at *TaElf3-B1* and *TaElf3-D1*, respectively. The unfavorable alleles associated with low TGW were detected at *TaSus2-2B*, *TaGW2-6A*, and *TaTGW6-4A*, while Hap-L associated with low grain numbers per spike at *TaMoc1-A1* were fixed in the diversity panel. The favorable alleles associated with low yellow-pigment contents (YPC) were fixed at *Psy-B1* and *Zds-D1*. The susceptible allele associated with Fusarium head blight gene (*Fhb1*) were fixed in diversity panel. Similarly, none of the accessions carried resistance alleles stem rust resistance gene (*Sr36/Pm6*), leaf rust resistance genes (*Lr37, Lr9, Lr67*, and *Lr47*) and eye-spot resistance gene (*CU8*). However, resistance allele for root lesion nematode resistance gene (*Rlnn1*) was present in 97.1% accessions (Table 2).

### Allelic Variation at Loci of Breeding Interest in SYN-DER

Several genes had more than two alleles, and only those genes are described here which have at least two alleles with more than 5% frequency. The minor allele frequency at 58 loci ranged from 5% (several alleles) to 47.6% (*1fehw3*). Allelic variation at these loci are described below according to their phenotypic associations (Table 1).

#### Wheat Adaptability and Developmental Related Genes

In total, 11 loci were categorized in this group (Table 1). At both *Rht* genes, wild-type alleles, *Rht-B1a* and *Rht-D1a*, were frequently present in 50.9 and 77.4% accessions, respectively. 1BL.1RS translocation was observed in 55 (26.4%) accessions. At photo-period response related genes, the photo-period insensitive alleles, *Ppd-A1a* and *Ppd-B1a*, were identified in 61.1 and 75.5% accessions, respectively. Sixteen accessions (7.7%) have GS-105 type *Ppd-A1a* allele with a 1,117 bp deletion in *Ppd-A1* and is likely to be transferred from the durum parents of synthetic hexaploid wheat (Table 1). Across three vernalization genes, the spring-type alleles had high frequency at *Vrn-A1* (45.2%), *Vrn-B1* (86.1%), and *Vrn-D1* (88.9%). The KASP assay TaBradi2g14790 was used to identify deletion of *Elf3-D1* gene associated with early flowering, and 46.1% of the accessions have gene deletion. Similarly, two paralogs of *Ppd1*, *PRR73-A1*, and *PRR73-B1* genes, were genotyped, and Hap-I was frequent (57.7%, 94.3%, respectively) at both loci (Table 1). Hap-I at *PRR73-A1* is associated with early flowering, while Hap-I at *PRR73-B1* is associated with delayed flowering (Zhang et al., 2016).

#### Grain Size and Weight Related Genes

In this category, 10 functional genes showed higher allelic variations, out of which two genes (*TaSus1-7A* and *TEF-7A*) had more than two alleles. At *TaGS-D1*, the favorable allele, *TaGS-D1a*, associated with high TGW was most frequent (82.2%). Similarly, higher frequency of favorable alleles was observed at *TaCwi-A1* (84.1%), *TaSus2-2A* (55.3%), *TaSus1-7A* (86.5%), *TaGS5-A1* (79.8%), *TaGW2-6B* (61.5%), *TaGS2-B1* (63.9%), and *TaGS1a* (58.1%), whereas unfavorable alleles associated with lower TGW were observed at *TaGASR* (88.9) and *TEF-7A* (65.8%) (Table 1).

#### Drought Adaptability Related Genes

Five genes related to drought adaptability showed higher allelic variation in SYN-DER. The favorable alleles, Hap-4A-C and *TaDreb-B1b*, showed higher frequency at *TaCwi-4A* and *TaDreb1* loci. Similarly, the favorable allele *COMT-3Ba* associated with high lignin contents under water-limited conditions was identified in 55.2% accessions. Almost equal allele frequency was observed at *1fehw3* locus related to water-soluble carbohydrate (WSC) contents in SYN-DER diversity panel (Table 1).

#### Pre-harvest Sprouting and Grain Color Related Genes

Seven genes related to grain color and dormancy showed higher allelic variation which also include three homeologous genes *TaMyb10* at A-, B-, and D-genomes. The alleles encoding white grain color were predominant at *TaMyb10-A1* and *TaMyb10-D1* loci, while red grain color encoding allele was predominant at *TaMyb10-B1*. The *Vp1-B1c* allele at *Vp1-B1* (69.2%), Rio-Balnco-type allele at *Phs1* (75.9%), and Zen-type allele at *TaMFT-A1* (85%) associated with pre-harvest sprouting tolerance were present with higher frequency (Table 1). At *TaSdr-B1*, the pre-harvest sprouting susceptibility allele, *TaSdr-B1b* had higher frequency.

#### End-Use Quality Related Genes

Two loci encoding high-molecular-weight glutenin subunits had relatively high frequency of A×1 (27.4%) and A×2^*^ (40.3%) at *Glu-A1* and D×5+Dy10 (50.4%) at *Glu-D1* which are associated with strong gluten contents and superior bread-making quality attributes. *WBM* is another newly identified bread-making quality gene, however, only 34 (16.3%) of the accessions have the favorable allele. Three major loci underpinning grain texture had high frequency of alleles associated with hard grain texture at *Pina-D1* (84.6%) and *Pinb-D1* (63.4%), whereas allele frequency was higher for soft grain texture at *Pinb-B2* (57.2%). Low YPC is a desirable trait for Chinese noodle and steamed bread for a brighter white color, whereas high YPC is favored for bread, yellow alkaline noodles, pasta, and some other products for higher content of carotenoids, and the alleles associated with YPC including *Psy-A1b, Psy-D1a*, and *Zds-A1a* were identified in 37.5, 94.7, and 53.8% of the accessions, respectively (Table 1).

#### Biotic Stress Resistance Genes

The frequency of adult-plant resistance gene *Lr34/Yr18* and *Sr2* was 54.3 and 3.3%, respectively. Similarly, two other leaf rust resistance genes *Lr14a* and *Lr21* were observed in 34.1 and 4.8% accessions, respectively. The alleles associated with virus resistance, *SbmP*, and soil born disease, *Cre8*, were detected in 9.6 and 22.6% of the accessions, respectively (Table 1).

### Genetic Diversity in the Diversity Panel Based on Functional Genes

**FIGURE 1:**
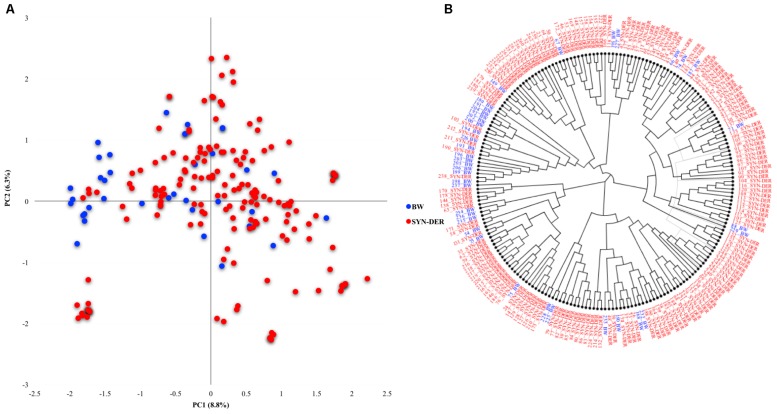
Genetic diversity in SYN-DER panel based on functional markers **(A)** the PCA scatter plot showing PC1 and PC2 on *x*-axis and *y*-axis, respectively, and **(B)** phylogenetic tree based on neighbor-joining approach is constructed.

Genetic diversity was estimated in the diversity panel based on the functional markers (Figure 1A). The accessions were categorized into synthetic-derivatives (those having *Ae. squarrosa* in their pedigree) and bread wheat advanced lines. The first two principal components explained 8.8 and 6.3% of the total variation, respectively. Most of the bread wheat accessions were separated on the PC2, and some were admixture within SYN-DER clusters. The phylogenetic tree corroborated the PCA analysis (Figure 1B), where some bread wheat accessions were in a distinct cluster, and remaining bread wheats clustered together with SYN-DER.

### Allelic Effects of Functional Genes in SYN-DER

All the KASP assays showing minor allele frequency >5% were used for marker-trait associations (MTAs) in the diversity panel using agronomic and biochemical traits under WW and WL conditions (Table 1). To avoid false associations, population structure matrix based on 500 unlinked SNP markers was used as co-variate. However, a relaxed criterion based on *P* < 0.05 was used to declare MTAs (Supplementary Table S3). Based on this criterion, 128 MTAs were observed, out of which 55 were associated with traits under WL conditions, 94 were associated with traits under WW conditions and 21 were associated across WW and WL conditions (Table 3). At stricter criterion of *P* < 0.01 the number of MTAs reduced to 24 under WL, 39 in WW and 3 across both water conditions. These include *Rht-D1, TaGS1a, Ppd-B1*, and *Vrn-A1* associated with TGW (Supplementary Table S3).

Five KASP assays for *Ppd1* homeologous genes were significantly associated with days to heading (DH), grain yield (GY), relative water contents (RWC), spike length (SL), and TGW. For *Ppd-A1* gene, GS105-type *Ppd-A1a* was associated with SL in WL condition and GY and spikelets per spike (SpPS) in WW conditions. The paralog of *Ppd1* gene, *PRR73-A1* was associated with DH and DM (Days to maturity) in WL conditions, GpS in WW condition, and tiller numbers (TN) in both conditions (Supplementary Table S3 and Table 3). The newly identified *Elf3-D1* and *TaMOT-D1* genes were associated with DEM, DH, DM, and HI in WL condition, while *TaMOT-D1* was also associated with DH and DM in WL condition.

**FIGURE 2:**
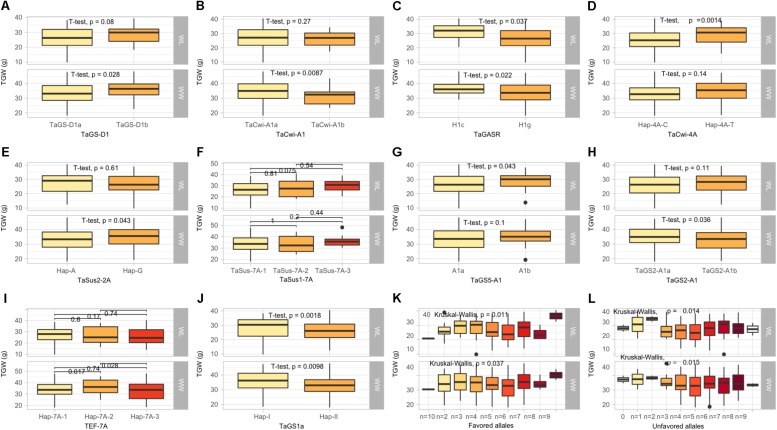
Box plots showing the allelic effects for grain size and weight related genes on thousand grain weight (TGW) in well-watered (WW), and water-limited (WL) conditions. In each box plot, **(A–J)**: the alleles/haplotypes of each gene are mentioned on *x*-axis, while phenotypic trait and its value (units) are mentioned on *y*-axis. The allelic effects of favorable alleles **(K)**, and unfavorable alleles **(L)** are shown. The allelic designations are provided in Table 1.

**FIGURE 3:**
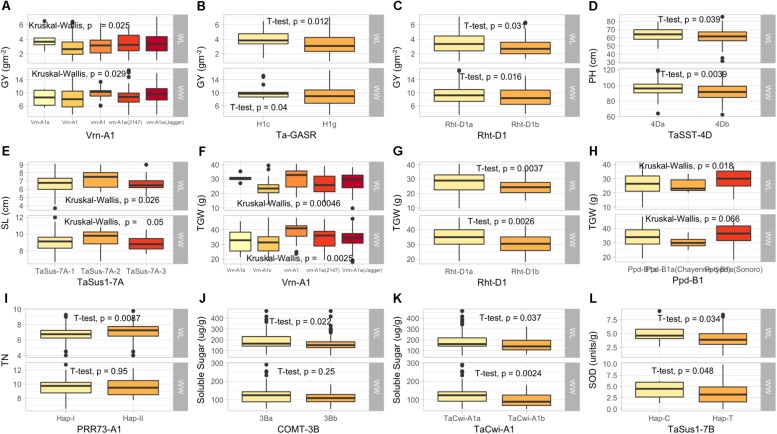
Box plots showing the allelic effects for drought tolerance, adaptability and flowering time related genes on grain yield (GY), plant height (PH), spike length (SL), tiller numbers (TN), soluble sugar (SS), and superoxide dismutase (SOD) in well-watered (WW), and water-limited (WL) conditions. In each box plot **(A–J)**: the alleles/haplotypes of each gene are mentioned on *x*-axis, while phenotypic trait and its value (units) are mentioned on *y*-axis. The allelic effects of favorable alleles **(K)**, and unfavorable alleles **(L)** are shown. The allelic designations are provided in Table 1.

The KASP assays for *TaSus1-7A* were associated with TGW and GY in WW condition, and SL in both conditions (Table 3 and Figures 2, 3). Wheat cell wall invertase gene, *TaCwi-4A*, was associated with DH and TGW in both water conditions, and GpS, PH, and proline in WW conditions (Table 3 and Figures 2, 3). Drought tolerance related gene such as *1fehw3* was associated with GY, PH, and SL in WW conditions. *Dreb1* was associated with canopy temperature (CT) and GpS in WW condition and HI in WL condition (Table 3). Surprisingly 1BL.1RS representing wheat rye translocation was associated with GpS, GY, SL, SOD, SpPS, and TGW in WW condition, however, no MTA was identified under WL conditions (Supplementary Table S3). *Rht-B1* was associated with GY, PH, and SL in WW condition (Supplementary Table S3) and GpS in both water conditions (Table 3). *Rht-D1* was associated with CT, GpS, RWC, and SOD in WW conditions (Supplementary Table S3), DEM and EL in WL-conditions (Supplementary Table S3), and GY, PH, and TGW in both water conditions (Table 3 and Figure 3). The KASP assay for *TaSST-4D* developed in this study was associated with biomass (BM) and TGW in WL conditions.

**FIGURE 4:**
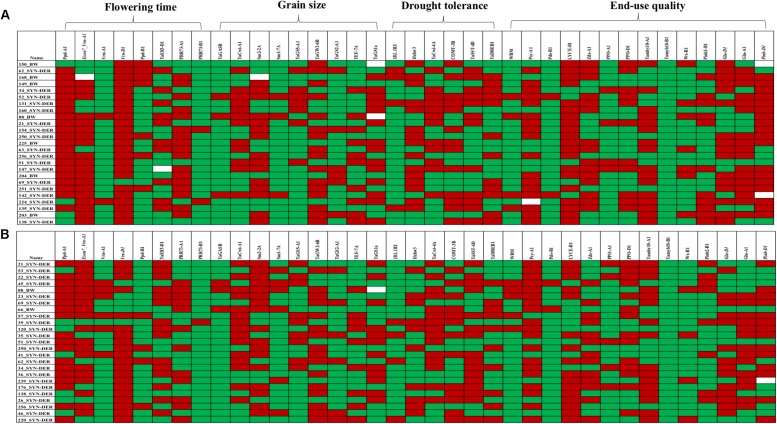
Graphical genotyping approach to show favorable (red) and unfavorable alleles (green) in top 20 high-yielding accessions under well-watered **(A)** and water-limited **(B)** conditions. Each row represents one accession and each column represents a functional gene. Gene groups are mentioned on top.

Several MTAs were identified for biochemical traits which provided novel insight into the confounding effect of functional genes on several biochemical traits and enzymatic activities induced by drought stress. Most importantly, *TaSus1-7B* was associated with superoxide dismutase (SOD) and *TaCwi-A1* was associated with total soluble sugar contents (SS) under WL conditions. A graphical genotyping approach was used to visualize the number of favorable alleles in 25 accessions in SYN-DER panel having the highest grain yield under WW (Figure 4A) and WL (Figure 4B) conditions.

## Discussion

The use of high-throughput KASP markers for functional genes provided valuable insight into the genetic architecture of synthetic-derived wheats for productivity, end-use quality, and disease resistance. This also helped to identify the favorable and unfavorable alleles of important breeding traits that are exhaustively selected and provide further opportunities to manipulate those alleles for wheat improvement. It had been challenging to practice molecular breeding in wheat despite the discovery and knowledge of huge array of functional genes for a range of important breeding traits (Liu et al., 2012). This was largely due to absence of high-throughput genotyping platform that can align with the breeding program to screen large breeding populations without compromising flexibility (Rasheed et al., 2017, Rasheed and Xia, 2019). Genomic studies using high-throughput genotyping assays like KASP had made it possible to genotype large populations at various loci within very short time (Rasheed et al., 2016a). Several recent studies used KASP markers to identify the allelic variation of functional genes in wheat cultivars from China (Rasheed et al., 2016a), United States (Grogan et al., 2016), and Canada (Perez-Lara et al., 2017).

Flowering time is one of the most important developmental traits for wheat adaptability and yield stability in target environments. Three genes controlling vernalization (*VRN1*), photoperiod response (*Ppd1*), and early flowering (*Elf3*) are known to be major determinants of flowering time optimization (Kamran et al., 2014). The alleles for spring growth habit were pre-dominantly observed at *VRN1* loci which is attributed to selections of the superior genotypes carried out in absence of vernalization conditions in field trials in Pakistan. It is mandatory to have all three recessive loci for complete vernalization requirement; none of the accessions had the three recessive alleles across *VRN1* homeologous loci. It has been known that *VRN1*, *Ppd1*, and *Elf3* have the extended roles beyond flowering time (Kamran et al., 2014). *VRN1* loci are known to increase number of spikelets (Whitechurch and Snape, 2003; Hailu and Merker, 2008), and final leaf number (Hay and Kirby, 1991). The association of allelic variation of *VRN1* and *Ppd1* homeologous loci with developmental traits like DH, DM, and yield related traits like GpS, GY, SL, SpPS, TGW, and TN was not unexpected. However, very little is known about the role of these genes under drought stress conditions, therefore our results have significant value for deployment of these genes under water-limited conditions. The *Vrn-A1* was significantly associated with GY and TGW, while *Ppd-B1* was associated with TGW under WL conditions. Previously, it was demonstrated (Ogbonnaya et al., 2017), that winter-type *VRN1* alleles significantly increased the heading time and decreased GY under heat stress conditions in bread wheat. Our results extended this information on the same role of *VRN1* alleles in drought stress conditions. Similarly, *Ppd1* roles are not confined to photo-period sensitivity, they also control modifications leading to spikes with more elaborated arrangements and increase number of grains producing spikelets (Boden et al., 2015). The association of *Ppd-A1* and *Ppd-B1* with yield-related traits including spikelets per spike was important finding and confirmed the role of *Ppd1* alleles in yield determination. The most important findings were that GS105-type *Ppd-A1a* alleles were retained in SYN-DER diversity panel at a relatively higher frequency and were associated with SL and spikelets per spike under WL conditions. These specific alleles are only present in durum wheat cultivars and are likely to have significantly higher expression for photo-period insensitivity. Because they are present in durum parents of synthetic hexaploid wheats, therefore this allele represented novel and potentially useful source of earliness in bread wheat. Because the diversity panel is fixed for major photo-period insensitive allele, *Ppd-D1a*, therefore these new variations from the durum source could help to further reduce flowering time in bread wheat. The *Ppd-1* genes were associated with other agronomic traits like GY, SL, SpPS, TGW, and TN in the diversity panel. These results are in agreement with previous findings (Boden et al., 2015; Rasheed et al., 2016b). The *PRR73* is a paralog of *Ppd-D1* in bread wheat, and it was reported that accessions having Hap-I at *PRR73-A1* and Hap-II at *PRR73-B1* were earlier in heading and taller under long day conditions than accessions having contrasting haplotype (Diaz et al., 2012). The association of *PRR73-B1* with PH and DH in SYN-DER diversity panel confirmed these finding, however, they were also associated with other traits under WL conditions. This indicated the importance of deploying PRR73 genes for drought tolerance by drought escape or maintaining assimilates during water-limited conditions.

Plant height is an important trait largely controlled by *Rht-B1* and *Rht-D1* genes. The important alleles *Rht-B1b* and *Rht-D1b* significantly reduce PH by 14–17%, decrease lodging, and increase harvest index (Rasheed et al., 2016a). The presence of *Rht-B1b* allele in 45% accessions and moderately high frequency of cultivar containing wild-type *Rht-D1a* allele was due to the fact that germplasm is derived from synthetic hexaploid wheats and mostly selected in drought conditions. The association of *Rht1* genes with many adaptive traits like DH, GpS, GY, PH, RWC, SL, SOD, and TGW indicated the pleiotropic effects of these two genes. Previous studies have shown very broad roles of *Rht* genes having pleiotropic effect on anther extrusion, a major trait in hybrid wheat production (Würschum et al., 2018), resistance against Fusarium head blight (Gosman et al., 2009), insect pest resistance (Emebiri et al., 2017), and grain quality. Therefore, modulating plant height by selecting appropriate *Rht* alleles according to target environment is not only important for pure-line breeding but can also assist in hybrid wheat breeding where tallness of male is required for effective production of hybrids (Würschum et al., 2018).

Grain size and weight is an important grain yield component, which have not been significantly increased as compared to grain numbers in many parts of the world. This indicated significant potential to exploit this trait component of grain yield in wheat. Until now, more than 15 genes have been cloned in wheat related to grain size and weight mainly using comparative genomics approaches and high gene co-linearity between wheat and other cultivated grass species like rice, maize, and barley (Nadolska-Orczyk et al., 2017). It is now well known through various experiments that drought stress at reproductive stage mainly restrict the spikelet fertility, thus reducing the number of grains (Ji et al., 2010), while drought stress at anthesis reduce the rate of resource mobilization from source to sink (grain) which ultimately reduce the grain size (Shen et al., 2003). Therefore, association of genes with grain size and grain number under WL provides further opportunities to use this information in developing drought tolerance varieties. In our panel, the favorable allele of *TaCwi* genes controls CWI enzyme which converts sucrose to glucose and fructose and expresses only in pollen, thus causes partial sterility in drought sensitive cultivars. As the both A- and D-genome homologs of *TaCwi* were positively selected in SYN-DER and associated with important agronomic traits, indicating the higher potential of SYN-DER in drought adaptability. Contrastingly, the unfavorable allele for another gene *TaMoc-A1*, a gene having an alleged role in spikelet development, was pre-dominant in the diversity panel and its favorable allele could be deployed for minor yield improvement under drought stress conditions. The other key genes either fixed in SYN-DER like *TaGW2-6A, TaSus2-2B*, and *TaCwi-D1* or having balanced allele frequency at *TaSus2-2A*, *TaGW2-6B*, and *TaGS1a* are very important to select accessions having maximum favorable haplotypes. It is likely that genes present on D-genome chromosomes like *TaCwi-D1* (5D) and *TaGS1a* (7D) could have new alleles which remained undetected because we only used functional markers for very well-known alleles.

Dehydration responsive element binding proteins, *DREB1*, have been induced by water stress, low temperature and salinity (Zhang et al., 2009). In this diversity panel, *TaDREB1* was associated with SL, GPS, and HI and was also previously associated with grain yield in Chinese wheats^28^. Similarly, Fructane 1 exohydrolase (*1-FEH*) is an ABA insensitive gene which is responsible for stable membrane and remobilizing water soluble carbohydrates (WSC) including fructan along with glucose and sucrose from stem to develop grains (Zhang et al., 2015). Rasheed et al. (2016a) developed a KASP marker for *1fehw3* gene, where Kauz-type allele has a very minor but significant effect on yield components, mainly TGW, in bread wheat (Howell et al., 2014). The presence of Kauz-type allele in 48% of the accessions and its association with GY, SL, and PH indicated the effectiveness to use this gene in wheat molecular breeding for drought tolerance.

The effect of drought on pollen fertility is irreversible and main cause of grain loss during WL conditions. Therefore, the storage carbohydrate accumulation in drought susceptible and tolerant cultivars depends on the genes for cell wall invertase, fructan biosynthesis genes in ovary and anthers (Shen et al., 2003). Since these genes tightly control sink strength and carbohydrate supply, therefore deployment of favorable alleles of these genes could maintain pollen fertility and grain number in wheat. The drought tolerance in SYN-DER diversity panel can be mainly attributed to the presence of favorable genes underpinning cell wall invertase (*TaCwi-A1* and *TaCwi-D1*) and enzymes related to remobilization of water soluble carbohydrates which ultimately strengthen the sink tissues during drought stress. The two KASP assays for *TaSST-A1* and *TaSST-D1* developed in this study are valuable tools for determining the water-soluble carbohydrates and their use in combination with *1fehw3* and *TaCwi* genes can enhance the selection accuracy of drought tolerant germplasm in marker-assisted breeding. Similarly, 1BL.1RS translocation also has significant yield advantage and has a positive impact on canopy water status (He et al., 2004). This translocation is widely used in breeding programs because it also provides resilience to biotic and abiotic stresses (Rasheed et al., 2016b). However, its positive or negative selection is based on the ultimate objective to develop cultivars, because this translocation is usually avoided due to the sticky dough characteristics of germplasm with 1BL.1RS translocation. 1BL.1RS translocation is present in 45% germplasm from CIMMYT, 22% from Turkey, 24% from China, 44% from Iran, 21% from Iran, and 17% from United States (Hailu and Merker, 2008).

The variability assessed for various quality encoding genes indicated the suitability of SYN-DER for variety of end-use quality characteristics. From results, it is clear that alleles associated with low PPO activity were higher at *Ppo-A1* and *Ppo-D1*, which is a desirable characteristic. Low PPO activity is preferred for fresh Asian noodles, and therefore, selection for the alleles *Ppo-A1b* and *Ppo-D1a* is recommended in Chinese wheat breeding program (Wang et al., 2009). High yellow pigment content is favored for durum wheat pasta, but is considered undesirable for Chinese steamed bread and white noodles (Huang and Morrison, 1988; He et al., 2008; Wang et al., 2009). Therefore, selection for the *Psy-A1b* and *Psy-B1c* is encouraged. Among 217 Chinese wheat cultivars, 135 had *Psy1-A1a* and 82 had *Psy1-A1b* alleles. Similarly, the frequency of *Psy-B1a* was 86, *Psy-B1b* was 95, *Psy-B1c* was 34, and *Psy-B1d* was 2 (He et al., 2009). At *Psy-D1*, 191 genotypes were *Psy1-D1a* and only two were *Psy1-D1g* in 193 Chinese wheat cultivars and advanced lines (He et al., 2009), indicating much less genetic diversity presented at the *Psy1-D1* locus.

A bright yellow color is desirable for yellow alkaline noodles, consumed in Japan and southeastern Asia; therefore, impairing LOX activity is desirable in wheat cultivars for use in these regions (Hessler et al., 2002). In China, however, a bright white to creamy color is required for Chinese style foods such as steamed bread and various Chinese noodles (He et al., 2008). Increasing LOX activity is therefore important in Chinese wheat breeding programs. In contrast, a bright yellow color is preferred for pasta, requiring a low LOX activity in durum wheat (*Triticum turgidum* L.) grains. Therefore, developing cultivars with lower LOX activity is an important objective in durum wheat breeding programs (Hessler et al., 2002; Carrera et al., 2007). Among these SYN-DER, almost all accessions have *TaLox-B1a* associated with high LOX activity, and this allele is desirable for Chinese-style foods but not for yellow alkaline noodles. However, the choice of *TaLox-B1a* or *TaLox-B1b* by breeders will depend on the projected end-use products of the breeding programs. Similar trend was observed for zeta-carotene desaturase alleles on two different loci. Alleles at *TaZds-D1* locus where *TaZds-D1b* alleles associated with low yellow-pigment content was fixed in diversity. In a previous study, *TaZds-D1b* allele was identified in four out of 217 Chinese varieties, while none of the advanced lines from CIMMYT carried this allele (Zhang et al., 2011). This indicated the *TaZds* gene at D-genome had very narrow genetic diversity in common wheat. The main limitation in comparing the diversity of such end-use quality related alleles is that most of the information is only available in Chinese wheat germplasm; hence it is important to reveal allelic variation for these genes in global wheat germplasm to build a knowledge based informatics resources in choosing appropriate candidates for wheat breeding.

Our work also included a comprehensive set of genes encoding biotic stress resistances to three rusts, powdery mildew, Fusarium head blight, and tan spot resistance. KASP assays for *Lr21* were adopted from previous reports, and was only present in 10 SYN-DER. The *Lr21* gene has exotic origin from *Ae. tauschii*. This indicated the potential to introduce these genes into wheat cultivars, as the leaf rust and stripe rust pathogens are widely avirulent to these genes globally (Sharma-Poudyal et al., 2013). Similarly, root lesion nematode resistance allele *Rlnn1* was present in all accessions of the diversity panel.

The large-scale gene characterization in SYN-DER panel was very useful allele repertoire for selection of accessions with favorable alleles. Field screening under WL conditions enhanced its value to use this information for a wide range of breeding objectives. It was mainly possible due to the availability of high-throughput KASP assays for functional genes in wheat partially reported in Rasheed et al. (2016a). However, still many new KASP assays have been developed by our groups and yet to be reported. This would enable wheat researchers to select germplasm during wheat breeding carrying alleles of choice to improve selection accuracy. The combination of several genes would create a specific haplotype which is important and efficient selection criteria compared to selection based on single genes. Wheat accessions with more favorable combination of genes (haplotypes) could be selected visually by graphical genotyping approach and would improve the selection accuracy in wheat breeding (van Eck et al., 2017). The graphical genotyping approach was used to visualize favorable allele frequencies in top yielding accessions in WW and WL conditions (Figure 4), which indicated slightly high frequency of favorable alleles of flowering time and drought tolerance in accessions having yield advantage under WL conditions. It is likely that several functional genes on D-genome chromosomes would carry novel alleles which remained undetected because we used KASP assays for well-known alleles at those loci. New approaches like target-enrichment sequencing of genes could be very effective for high-throughput SNP discovery in novel germplasm like SYN-DER derived from the wild species (Pankin et al., 2018).

The diversity panel was highly divergent for functional genes and this provided a set of target genes which could be manipulated to further fine-tune the expression of important agronomic traits. Several accessions were selected based on the combination of favorable alleles, i.e., SD89 has 30 favorable alleles and SD36 carries 27 favorable alleles of important traits. We also provided new insight on effects of functional genes for important biochemical traits under drought stress conditions which could be important for developing drought tolerant cultivars.

## Author Contributions

AR, ZH, and XX designed the experiments. MK, ZM, FA, AG, and AS conducted the field experiments. MK and ZA did the genotyping work. AR analyzed the data. AR and MK wrote the manuscript. ZH, XX, and RA edited and reviewed the manuscript.

## Conflict of Interest Statement

The authors declare that the research was conducted in the absence of any commercial or financial relationships that could be construed as a potential conflict of interest.
